# Portable solid-state sensor for therapeutic monitoring of an antineoplastic drug; vinblastine in human plasma†

**DOI:** 10.1039/d0ra07070j

**Published:** 2020-11-24

**Authors:** Maha Mohammed Galal, Ahmed Sayed Saad

**Affiliations:** Analytical Chemistry Department, Faculty of Pharmacy, Cairo University Kasr El-Aini St, PO 11562 Cairo Egypt maha.galal@pharma.cu.edu.eg ahmed.bayoumy@pharma.cu.edu.eg +201117486474 +201004009443; Pharmaceutical Chemistry Department, School of Pharmacy and Pharmaceutical Industries, Badr University in Cairo (BUC) Badr City 11829 Cairo Egypt

## Abstract

During cancer treatment, doses must be carefully administered and monitored to guarantee efficacy and minimize side-effects. A potentiometric sensor was developed for the direct real-time assay of a widely used antineoplastic drug (vinblastine (VB)) in plasma samples. Membrane cocktails were drop-casted over a glassy-carbon electrode coated with a lipophilic conducting polymer (polyaniline). The study investigated five cation exchangers, five plasticizers (of different polarities and dielectric constants), and four ionophores with different physicochemical characters on the sensor performance. The study substantiates a data-driven selection of the optimum membrane recipe. The latter included sodium tetraphenylborate as an ion exchanger, dioctylphthalate as a plasticizer, and hydroxypropyl-β-cyclodextrin as ionophore. The membrane proved a near-Nernstian slope of 37.5 mV per decade, a LOQ of 2.99 × 10^−6^ M, and a stable fast response. The selectivity study proved poor responses to common physiological ions. The developed sensor was used for the determination of VB in its pure powder form, marketed formulation, and plasma samples. The fast and direct sensor response enables a wide range of applications in quality control laboratories and clinical studies.

## Abstract 2

Potentiometric glassy carbon electrode for determination of vinblastine.

## Graphical abstract

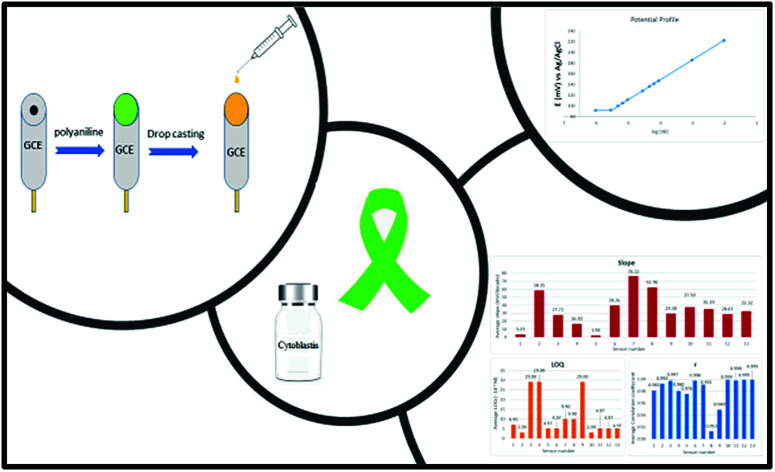

## 1 Introduction

Cancer is the uncontrollable growth and spread of abnormal cells throughout the body organs. WHO ranks cancer as the second leading cause of death worldwide. Cancer claimed 9.6 million lives (one-sixth of the deaths) in 2018. The uneven distribution of mortalities reinforces the conclusion that early diagnosis and quality treatment contribute to significantly higher survival rates in developed countries than developing countries.^1^

Vinblastine is a well-tolerated inexpensive chemotherapeutic alkaloid extracted from Madagascar periwinkle known as *Catharanthus roseus* or *Vinca rosea*. The plant produces a similar chemotherapeutic alkaloid called vincristine that slightly differs in chemical structure,^2^ (ESI Fig. S1†). Vinblastine is less expensive, and 1000 times more abundant in *Vinca rosea* than vincristine.^3^

Both—vinblastine and vincristine—are cell cycle-specific alkaloids that bind to the microtubular protein, tubulin, to prevent cell division during the metaphase. They inhibit purine synthesis, citric acid cycle, and urea formation, affect amino acid metabolism and DNA synthesis, and exert an immunosuppressive action.^4,5^ Both drugs are used in the treatment of acute lymphoblastic leukemia Hodgkin's lymphoma, and other lymphomas.^6^

Vinblastine is a dimer formed by coupling vindoline and catharanthine alkaloids in *Vinca rosea*. Vincristine is produced through vinblastine oxidation. Both vinblastine and vincristine are present in relatively small amounts in the plant. This justifies the high production costs that burden the financials of individuals, communities, and health care systems. Scientists have tried chemical,^3^ biotechnological,^7–9^ or microbiological^10–12^ pathways to maximize vinblastine and vincristine yields.

Vinblastine is commonly administrated in the form of its sulfate salt prepared as 1 mg mL^−1^ in 0.9% sodium chloride solution injected intravenously. The rapid body clearance, large distribution volume, and low plasma concentration of vinblastine necessitate a rapid, sensitive, and selective analytical method to achieve reliable pharmacokinetic studies.^13,14^

Different methods have been reported in the literature for the determination of vinblastine including spectrophotometry,^15,16^ thin layer chromatography,^17^ high-performance liquid chromatography,^18–26^ capillary electrophoresis,^27–31^ and voltammetric methods.^32–37^

As far as the authors' knowledge, no potentiometric sensor is reported in the literature for the determination of vinblastine. Therefore, it was our concern to develop a simple, sensitive, and selective method for the quantitative analysis of such a life-saving drug, taking advantage of the superior inherent benefits of ion-selective electrode-potentiometry such as simplicity, portability, rapidity, and lower energy consumption. The developed method intends to validate a sensor for vinblastine assay in pure form, pharmaceutical dosage form, and plasma samples.

## 2 Experimental

### 2.1 Apparatus

A Jenway potentiometer (3310, UK) was used to record the potential difference between the working electrodes against an Orion (900200, UK) double junction Ag/AgCl reference electrode. A Daihan (MSH-20D, Korea) hot plate and stirrer and a Jenway (924051, UK) glass electrode were used.

### 2.2 Chemicals and reagents

Standard vinblastine sulfate (VB) working standard (99.65% purity) was kindly obtained from the National Organization of Drug Development and Research. Analytical grade reagents and solvents were employed in the study. Poly(vinyl)chloride (PVC), phosphomolybdic acid (PM), phosphotungstic acid (PT), sodium tetraphenylborate (TPB), ammonium reineckate (RK), potassium tetrakis (TKS), nitrophenyl octyl ether (NPOE), dioctyl phthalate (DOP), dibutyl phthalate (DBP), dibutyl sebacate (DBS), nitrophenyl phenyl ether (NPPE), hydroxypropyl-β-cyclodextrin (HPBCD), β-cyclodextrin (BCD), carboxymethyl-β-cyclodextrin (CMBCD), calix-[8]-arene (CX8) and tetrahydrofuran (THF) were obtained from Sigma-Aldrich. Sodium hydroxide, potassium dihydrogen phosphate, and hydrochloric acid were obtained from (El-Nasr Pharmaceutical Chemical Company, Cairo, Egypt). A 50 mM KH_2_PO_4_ solution adjusted to pH 4.0 ± 0.1 was used as a buffer. Plasma samples were purchased from Vacsera Co. (Giza, Egypt).

### 2.3 Standard solutions

Stock standard solution 1.00 × 10^−2^ M VB was prepared in bi-distilled water. Serial dilutions of the stock standard solution were carried out to prepare VB working standard solutions in the range 1.00 × 10^−2^ to 1.00 × 10^−6^ M using the phosphate buffer pH 4.0 as a diluent.

### 2.4 Procedure

#### 2.4.1 Membrane cocktails preparation

Different membrane cocktails were prepared in 5 mL volumetric flasks by transferring accurately weighed amounts of the membrane components including PVC, ion-exchanger (PM, PT, TPB, RK, and TKS), plasticizer (NPOE, DOP, DBP, DBS, and NPPE), and ionophore (HPBCD, BCD, CMBCD, and CX8). The composition of each sensor is supplied in ESI Table S1.† The components were dissolved in THF and the volume was completed to the mark using THF.

##### 2.4.1.1 Sensor assembly

The surface of the glassy carbon electrode was polished using Al_2_O_3_ based slurry, sonicated in deionized water for 15 minutes, and rinsed with acetone to complete the polishing step. Polyaniline (PANI) was electro-polymerized—onto the polished glassy carbon surface—in a three-electrode electrochemical cell by cyclic voltammetry. The reference, auxiliary (Pt), and working glassy carbon electrodes were placed in a solution containing 0.45 M aniline and 1 M HCl. The potential was cycled between −0.2 V and 0.8 V in 2 mV steps at a 50 mV s^−1^ scan rate for 5 successive cycles. A micropipette was used to drop cast an accurate volume of the membrane cocktails over the PANI coated glassy carbon electrode surface.

#### 2.4.2 Sensor optimization

The composition of the VB sensor was optimized as a function of the response parameters: Nernstian slope, linearity, and quantification limit.

### 2.5 Calibration of the VB sensor

The potential difference developed between each of the prepared sensors and a double junction silver/silver chloride reference electrode was measured in the prepared VB working standard solutions containing different concentrations of VB. Calibration graphs were plotted to correlate the measured potential difference to the logarithm function of the molar concentration of VB. Regression equations were computed to deduce Nernstian slope, linear range, and linearity.

### 2.6 Limit of detection

IUPAC recommendations^38^ were followed to calculate the LOD at the intersection of the two straight segments at the low concentration side of the calibration curves.

### 2.7 Response time

The time required for the sensor to reach a final stable response (±1.0 mV) was estimated dynamically after successive additions from VB standard solution.

### 2.8 Effect of pH

The optimized sensor was used to monitor the difference in potential within 1 × 10^−4^ and 1 × 10^−5^ M VB solutions after altering their pH using small increments of 0.1 M HCl and 0.1 M NaOH. A potential *vs.* pH curve was plotted using the simultaneously recorded pH and potential measurements following each addition.

### 2.9 Sensor selectivity

The separate solution method (SSM) recommended by the IUPAC^39,40^ was followed to calculate the potentiometric selectivity coefficient *K*^pot^_VB,int_ for different cationic contaminants (Na^+^, K^+^, Zn^2+^, Mn^2+^, Pb^2+^, Co^2+^, Mg^2+^, Cu^2+^, Fe^2+^, Ca^2+^, Ni^2+^, NH_4_^+^ and Ba^2+^).1
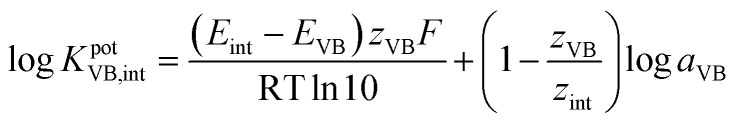
where *E*, *a*, and *z* are the potential difference, activity, and charge of VB and interfering species (int), respectively.

### 2.10 Application

Cytoblastin® vial is labeled to contain 1 mg mL^−1^ vinblastine sulfate. A solution having a concentration of 1.1 × 10^−5^ M VB was prepared by suitable dilution of the dosage form then the standard addition technique was carried out. The concentration of the final solution was calculated using the following simplified equation.2
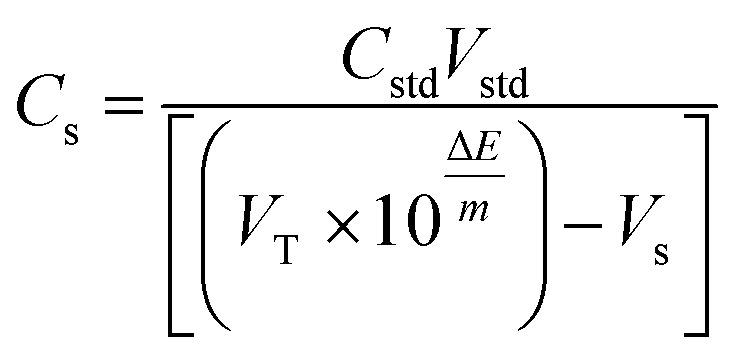
where, *C*_s_ and *C*_std_ are the molar concentrations of the sample and the standard solutions, *V*_s_, *V*_std_ and *V*_T_ are the sample, standard and total solution volume in mL, Δ*E* is the change in potential after addition of the standard solution in mV and *m* is the slope of the calibration curve for the employed sensor in mV per decade.

Validation of the sensor's performance was carried out in the studied matrices (dosage form and plasma) through the back determination of the concentration of spiked standard using the aforementioned standard addition technique.

## 3 Results and discussion

The cornerstone in potentiometric sensor development is the proper selection of the experimental conditions and sensor matrix ingredients (type and amount) that achieve peak performance. At which, the ingredients work altogether in synchronized harmony to achieve the desired response. During the optimization study, one should consider all factors that affect the response. Based on our past experiences, conditions such as type and amount of the ion-exchanger, plasticizer, and ionophore within the PVC cocktail, as well as the experimental conditions such as pH, directly affect the sensor performance.

It is necessary to define the optimum sensor performance before starting the optimization process. An optimum sensor develops a stable, linear, Nernstian response for the change in analyte concentration, within a short response time with sufficient sensitivity and selectivity. During the optimization process, calibration graphs were constructed and sensor performance parameters including slope, LOQ, LOD, correlation coefficient, and selectivity were calculated according to the IUPAC recommendations.^38^

### 3.1 Ion exchanger

Lipophilic ion-exchangers regulate the membrane permselectivity, by allowing selective extraction of the analyte ion and excluding the co-extraction of the interfering ions from the sample solution. Their lipophilic nature creates a preferential solubility within the PVC membrane matrix and prevents the ion-exchangers from leaching into the aqueous sample solution.^41^ The confined ionic sites—within the membrane matrix—not only induce a selective response for the oppositely charged analyte but also act to reduce the membrane resistance and the ionic interference from similarly charged ions within the sample solution (Donnan exclusion effect).

Sensors 1, 2, 3, 4, and 5 contain ion exchangers PM, PT, TPB, RK, and TKS, respectively. The response of the five sensors was recorded for the change in VB concentration (ESI Fig. S2†). Sensors 1, 4, and 5 expressed sub-Nernstian slopes. Sensor 2 with PT as an ion-exchanger showed a super-Nernstian slope. Sensor 3 containing TPB as an ion exchanger expressed a near-Nernstian slope for the divalent VB cation.

**Fig. 1:**
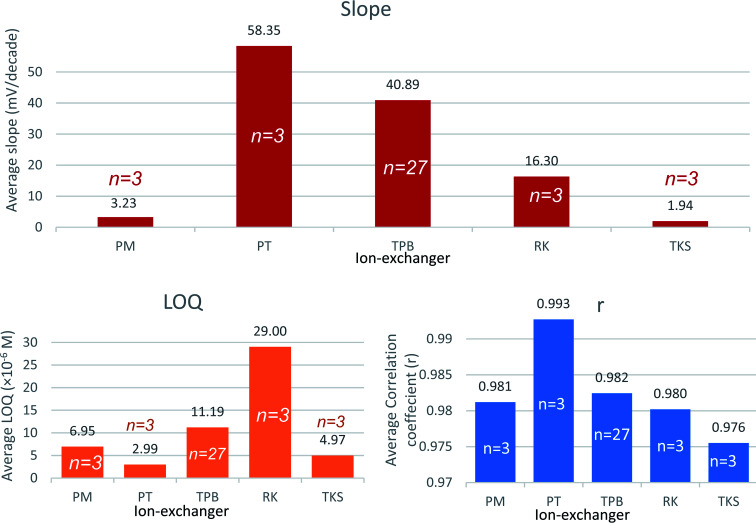
Average slope, LOQ, and correlation coefficient (*r*) obtained using the studied ion-exchangers phosphomolybdic acid (PM), phosphotungstic acid (PT), sodium tetraphenylborate (TPB), ammonium reineckate (RK), potassium tetrakis (TKS).

Pooled ion-exchanger performance data (Fig. 1) shows that sensor 3 was generally able to approach the divalent Nernstian slope with an acceptable quantification limit and correlation coefficient. This indicates the preferential ability of VB to form ion-pair with TPB (sterically favored). Therefore, TPB was selected as an ion-exchanger through the study.

Super-Nernstian slopes may be attributed to the formation of the monovalent complex ions [VB–X]^+^ between the ion-exchanger (X^−^) and the divalent vinblastine (VB^2+^).

### 3.2 Plasticizer

The type and amount of plasticizer included within the membrane matrix control the mobility of the membrane constituents thus affects the membrane resistance. Plasticizers play a role in membrane selectivity by influencing the standard free energy of the ions. They also control the ion-pair formation, thus affect the slope and the response time. Plasticizers determine the membrane lifetime through the direct control of the membrane polarity and thus limit the exudation of the membrane constituents into the aqueous sample solution.^42^

Sensors 3, 6, 7, 8, and 9 contain 5 different plasticizers: NPOE, DOP, DBP, DBS, and NPPE, respectively. Based on reports and recent work the PVC to plasticizer ratio was held at 1 : 2.^43,44^

**Fig. 2:**
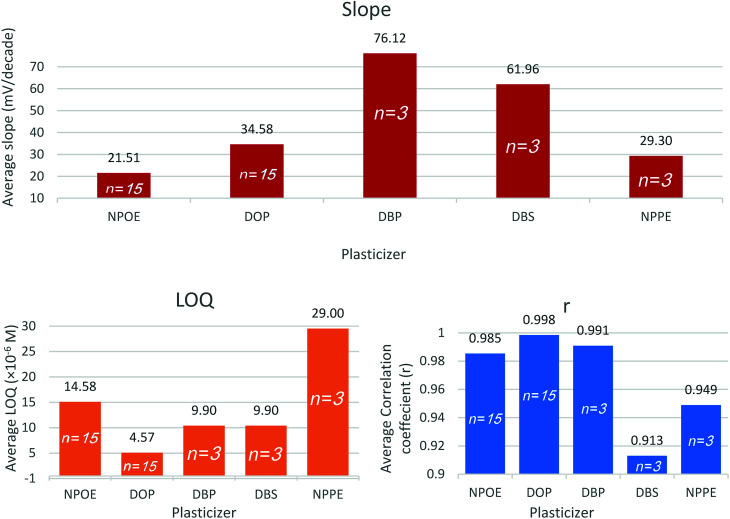
Average slope, LOQ, and correlation coefficient (*r*) obtained using the studied plasticizers nitrophenyl octyl ether (NPOE), dioctyl phthalate (DOP), dibutyl phthalate (DBP), dibutyl sebacate (DBS), nitrophenyl phenyl ether (NPPE).

Both DBS and DBP sensors expressed a super-Nernstian slope. DOP as a plasticizer (sensor 6) demonstrated the lowest quantification limit and highest correlation coefficient. Pooled plasticizer performance data substantiate the latter findings (Fig. 2). The results indicated that DOP modifies the membrane characteristics to enable faster exchange kinetics with VB ions.

The relatively low polarity of DOP is advantageous in plasma application since it reduces the deposition of charged species such as proteins over the surface of the sensor.^42^

### 3.3 Ionophore

Ionophores are lipophilic host molecules that possess polar function groups for ionic recognition. They remain retained within the lipophilic membrane owing to their lipophilic character. Recognition principles differ according to the ionophore structure, but they all share the formation of a thermodynamically strong but kinetically weak complex with the analyte ion in the membrane. Thus, selectivity is partially controlled by the complex formation constant as well as the standard Gibb's free energy of ion transfer between the aqueous sample and the membrane. Successful ionophores selectively bind to the analyte ions and suppress competition from interfering ions, and keep constant analyte concentration within the membrane phase, thus potential changes are exclusively due to the analyte activity in the sample solution.^41,42,45^

**Fig. 3:**
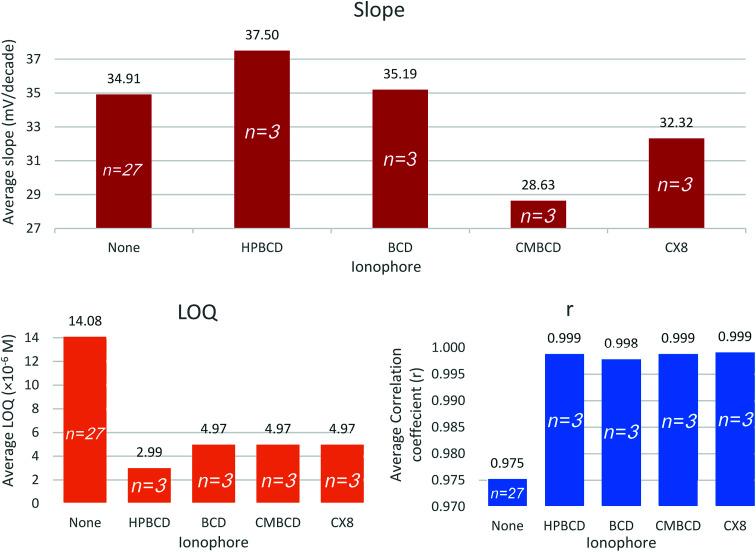
Average slope, LOQ and correlation coefficient (*r*) obtained using the studied ionophores hydroxypropyl-β-cyclodextrin (HPBCD), β-cyclodextrin (BCD), carboxymethyl-β-cyclodextrin (CMBCD), calix-[8]-arene (CX8).

Sensors 10, 11, 12, and 13 contain HPBCD, BCD, CMBCD, and CX8, respectively. The presence of ionophores significantly improves the quantification limit and linearity. Generally, all ionophores were able to improve the slopes, decrease the quantification limits, and increase the linearity coefficients (Fig. 3 and ESI Fig. S2†). However, HPBCD was able to decrease the quantification limit down to 60% compared to the other three ionophores as well as the ionophore free sensor (sensor 6) (Fig. 3 and ESI Fig. S2†). Vinblastine forms a more stable thermodynamically favored complex with HPBCD than other studied ionophores. The large carbon skeleton, extended conjugation, polar hydroxyl and amino groups, and the two positive centers in the chemical structure of VB offers several sites for non-covalent interaction with the ionophore (*e.g.* hydrogen bonding, van der Waals interaction, and hydrophobic interaction).

The optimized sensor recipe included 32.3% PVC, 1.0% TPB, 64.7% DOP, and 2.0% HPBCD.

### 3.4 Effect of pH

The potential was monitored whilst the pH deliberately and gradually fluctuated. The procedure was repeated using two different concentrations of VB (10^−4^ and 10^−5^ M). The sensor demonstrated a stable response within the pH range of 3 to 6. The response gradually decreases beyond pH 6 owing to the deprotonation of the weakly basic analyte (p*K*_a_ of VB is 8.86) forming the unionized vinblastine base (ESI Fig. S3†). At pH < 3, protons in the solution compete with VB for the membrane negatively charged sites leading to a deteriorated response for VB.

### 3.5 Dynamic response time

The optimized sensor demonstrated a rapid Nernstian response for the deliberate changes in the concentration of VB (5 × 10^−6^ to 5 × 10^−3^ M). Stable responses (±1 mV of the equilibrium potential) were attained within 2–5 seconds. The sensor spends a relatively long time to reach the equilibrium potential at lower VB concentrations than it does at higher concentrations. The overall response time of the sensor for VB is such rapid to enable real-time assay of VB in pharmacokinetic studies, provided that the sensor expresses sufficient selectivity for VB in plasma (ESI Fig. S4†).

### 3.6 Selectivity

The sensor selectivity was assessed for commonly encountered cations in plasma and dosage form. The potentiometric selectivity coefficients calculated using the separate solution method show that the sensor demonstrated higher selectivity for VB relative to the examined ions. Interestingly, the sensor exhibited higher selectivity for VB than its structurally similar analog vincristine sulfate (ESI Table S2†).

**Fig. 4:**
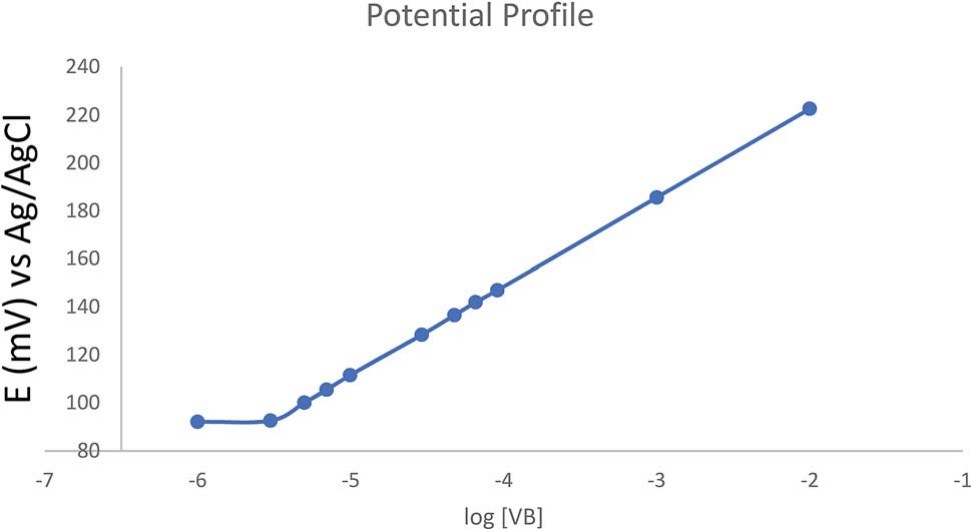
Potential profile of the optimized sensor against different concentrations of vinblastine in buffer pH 4.

The potential profile for the optimized sensor containing TPB as ion-exchanger, DOP as a plasticizer, and HPBCD as ionophore was scanned in phosphate buffer pH 4 (Fig. 4).

The ICH validation parameters (accuracy, repeatability, reproducibility, linearity) were computed for the optimized sensor under the optimized conditions (Table 1).

**Table 1:** Assay validation parameters of the optimized sensor

Parameter	Value^a^
Slope (mV per decade)	37.50 ± 0.315
Intercept (mV)	296.74 ± 1.209
Correlation coefficient	0.9999
Concentration range (M)	2.99 × 10^−6^ to 1.00 × 10^−2^
Detection limit (M)	2.89 × 10^−6^
Response time (s)	2–5
Working pH range	3–6
Lifetime (weeks)	2
Accuracy (mean recovery ± % RSD)	99.96 ± 1.739

**Precision**	
(a) Repeatability^b^ (% RSD)	0.770
(b) Reproducibility^c^ (% RSD)	1.956

a Average of three determinations.

b Average of three concentrations repeated three times within the same day.

c Average of three concentrations repeated three times on three successive days.

Owing to the divalent nature of VB, we did not manage to use the direct method for the assay. Since divalent ions are more labile to changes in the ionic strength of the sample solution than do monovalent ions. Instead, the standard addition technique was adopted for VB assay in Cytoblastine® vials and spiked plasma samples. The change in the potential of the sample solution was recorded after the addition of a relatively smaller volume of standard VB solution to minimize the fluctuations in the junction potential. Results were validated by back determination of VB standard concentrations spiked to Cytoblastine® samples and plasma (ESI Table S3†).

A battery-powered portable potentiometer can directly measure the difference in potential between the developed sensor and a reference electrode in the sample solution without sample preparation or derivatization steps.

## 4 Conclusion

The work introduces a potentiometric sensor for the assay of VB. The sensor was optimized to reach the optimal sensor composition. The sensor included tetraphenylborate as ion-exchanger and dioctyl phthalate as a plasticizer. Four hosting ionophores were used to evaluate the effect of host–guest chemistry on the sensor response. Hydroxypropyl-β-cyclodextrin minimized the quantification limit and improved the correlation coefficient. The optimized sensor demonstrated a rapid response for the change in vinblastine concentration within the concentration range 2.99 × 10^−6^ M to 1.00 × 10^−2^ M with a slope of 37.5 mV per decade. The sensitive, selective, fast, stable response of the sensor enabled VB assay in plasma samples. The absence of sample preparation and treatment steps eliminates the need for additional chemicals and solvents and reduces the impact of the developed method on the environment.

## Conflicts of interest

There are no conflicts to declare.

## Supplementary Material

RA-010-D0RA07070J-s001

## References

[cit1] World Health Organization , Cancer, https://www.who.int/news-room/fact-sheets/detail/cancer, accessed, 14 April 2020

[cit2] Aniszewski T. , Alkaloids-Secrets of Life: Alkaloid Chemistry, Biological Significance, Applications and Ecological Role, Elsevier, 2007

[cit3] Kumar A. (2016). International Journal of Medicine and Pharmaceutical Sciences.

[cit4] Prendergast G. C. and Jaffee E. M., Cancer Immunotherapy, Elsevier, 1st edn, 2007

[cit5] Aniszewski T. , Alkaloids – secrets of life, alkaloid chemistry, biological significance, applications and ecological role, Elsevier, 2007

[cit6] Dowd F. J. , Johnson B. S. and Mariotti A. J., Pharmacology and Therapeutics for Dentistry, Elsevier, 2017

[cit7] Barrales-Cureño H. (2015). Biotecnol. Apl..

[cit8] Ta H. S., El-Bahr M. K., Seif-EL-Nasr M. M. (2008). Appl. Sci. Res..

[cit9] El-Sayed M., Verpoorte R. (2007). Phytochem. Rev..

[cit10] Almagro L., Fernández-Pérez F., Pedreño M. A. (2015). Molecules.

[cit11] Ayob F. W., Simarani K., Zainal Abidin N., Mohamad J. (2017). Microb. Biotechnol..

[cit12] Kumar A., Patil D., Rajamohanan P. R., Ahmad A. (2013). PLoS One.

[cit13] Owellen R. J., Hartke C. A., Hains F. O. (1977). Cancer Res..

[cit14] Rahmani R., Zhou X.-J. (1993). Cancer Surv..

[cit15] Nagaraja P., Vasantha R. A., Yathirajan H. S. (2002). J. AOAC Int..

[cit16] Jeewantha H. A., Ivanovich S. A., Mihailovich K. P. (2017). Int. J. Pharm. Pharm. Sci..

[cit17] Paci A., Mercier L., Bourget P. (2003). J. Pharm. Biomed. Anal..

[cit18] Singh D. V., Maithy A., Verma R. K., Gupta M. M., Kumar S. (2000). J. Liq. Chromatogr. Relat. Technol..

[cit19] Gupta M. M., Singh D. V., Tripathi A. K., Pandey R., Verma R. K., Singh S., Shasany A. K., Khanuja S. P. S. (2005). J. Chromatogr. Sci..

[cit20] Kosjek T., Dolinšek T., Gramec D., Heath E., Strojan P., Serša G., Čemažar M. (2013). Talanta.

[cit21] Zhang L., Gai Q.-H., Zu Y.-G., Yang L., Ma Y.-L., Liu Y. (2014). Chin. J. Nat. Med..

[cit22] Fabrizi G., Fioretti M., Mainero Rocca L. (2016). Biomed. Chromatogr..

[cit23] Abdul Rahim R., Ahmad N. H., Al Azzam K. M., Mat I. (2018). Adv. Pharm. Bull..

[cit24] Jeong W. T., Bin Lim H. (2018). J. Chromatogr. B: Anal. Technol. Biomed. Life Sci..

[cit25] Gao S., Tao Z., Zhou J., Wang Z., Yun Y., Li M., Zhang F., Chen W., Miao Y. (2018). J. Anal. Methods Chem..

[cit26] Guichard N., Fekete S., Guillarme D., Bonnabry P., Fleury-Souverain S. (2019). J. Pharm. Biomed. Anal..

[cit27] Chu I., Bodnar J. A., White E. L., Bowman R. N. (1996). J. Chromatogr. A.

[cit28] Barthe L., Ribet J.-P., Pélissou M., Degude M.-J., Fahy J., Duflos A. (2002). J. Chromatogr. A.

[cit29] Sidorova A. A., Yaroshenko D. V., Murashko E. A., Grigor'ev A. V. (2013). J. Anal. Chem..

[cit30] Abd El-Hady D., Albishri H. M., Rengarajan R. (2015). Biomed. Chromatogr..

[cit31] Guichard N., Ogereau M., Falaschi L., Rudaz S., Schappler J., Bonnabry P., Fleury-Souverain S. (2018). Electrophoresis.

[cit32] Temizer A. (1986). Talanta.

[cit33] Brett A. M. O., Grazina M. M. M., Macedo T. R. A., Raimundo D. (1994). Electroanalysis.

[cit34] Haque I. U. and Saba H., in ECS Transactions, ECS, 2009, vol. 16, pp. 3–23

[cit35] Cavalheiro É. T. G., Brett C. M. A., Oliveira-Brett A. M., Fatibello-Filho O. (2012). Bioanal. Rev..

[cit36] Haghshenas E., Madrakian T., Afkhami A., Saify Nabiabad H. (2017). Anal. Bioanal. Chem..

[cit37] Kurbanoglu S., Bakirhan N. K., Gumustas M., Ozkan S. A. (2019). Crit. Rev. Anal. Chem..

[cit38] P. Press , Pure Appl. Chem., 1976, vol. 48, pp. 127–132

[cit39] IUPAC R. , Pure Appl. Chem., 2000, vol. 72, pp. 1851–2082

[cit40] IUPAC R. , Pure Appl. Chem., 2000, vol. 72, pp. 1851–2082

[cit41] Bakker E. , in Reference Module in Chemistry, Molecular Sciences and Chemical Engineering, Elsevier, 2018

[cit42] Bakker E., Bühlmann P., Pretsch E. (1997). Chem. Rev..

[cit43] Moody G. J., Oke R. B., Thomas J. D. R. (1970). Analyst.

[cit44] Yehia A. M., Saad A. S., Tantawy M. A. (2020). J. Pharm. Biomed. Anal..

[cit45] Bakker E., Pretsch E. (2002). Anal. Chem..

