# Supplementary material for: Portable solid-state sensor for therapeutic monitoring of an antineoplastic drug; vinblastine in human plasma
Source: RSC Adv. 2020 Nov 24;10(70):42699–705. doi: 10.1039/d0ra07070j (PMC9058239; doi:10.1039/d0ra07070j)
Supplement: RA-010-D0RA07070J-s001 [file RA-010-D0RA07070J-s001.pdf]

Electronic Supplementary Material (ESI) for RSC Advances.
This journal is © The Royal Society of Chemistry 2020

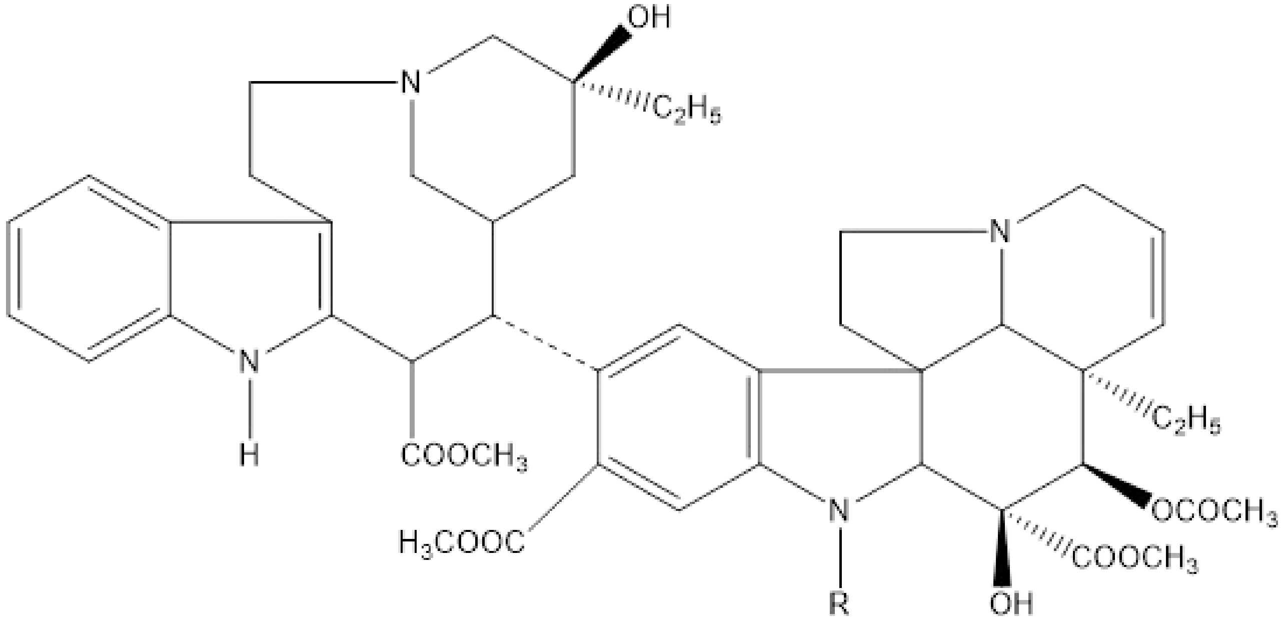

Vinblastine: R = CH_3

Vincristine: R = CHO

Supplementary Figure S1. Chemical structure of vinblastine and vincristine.

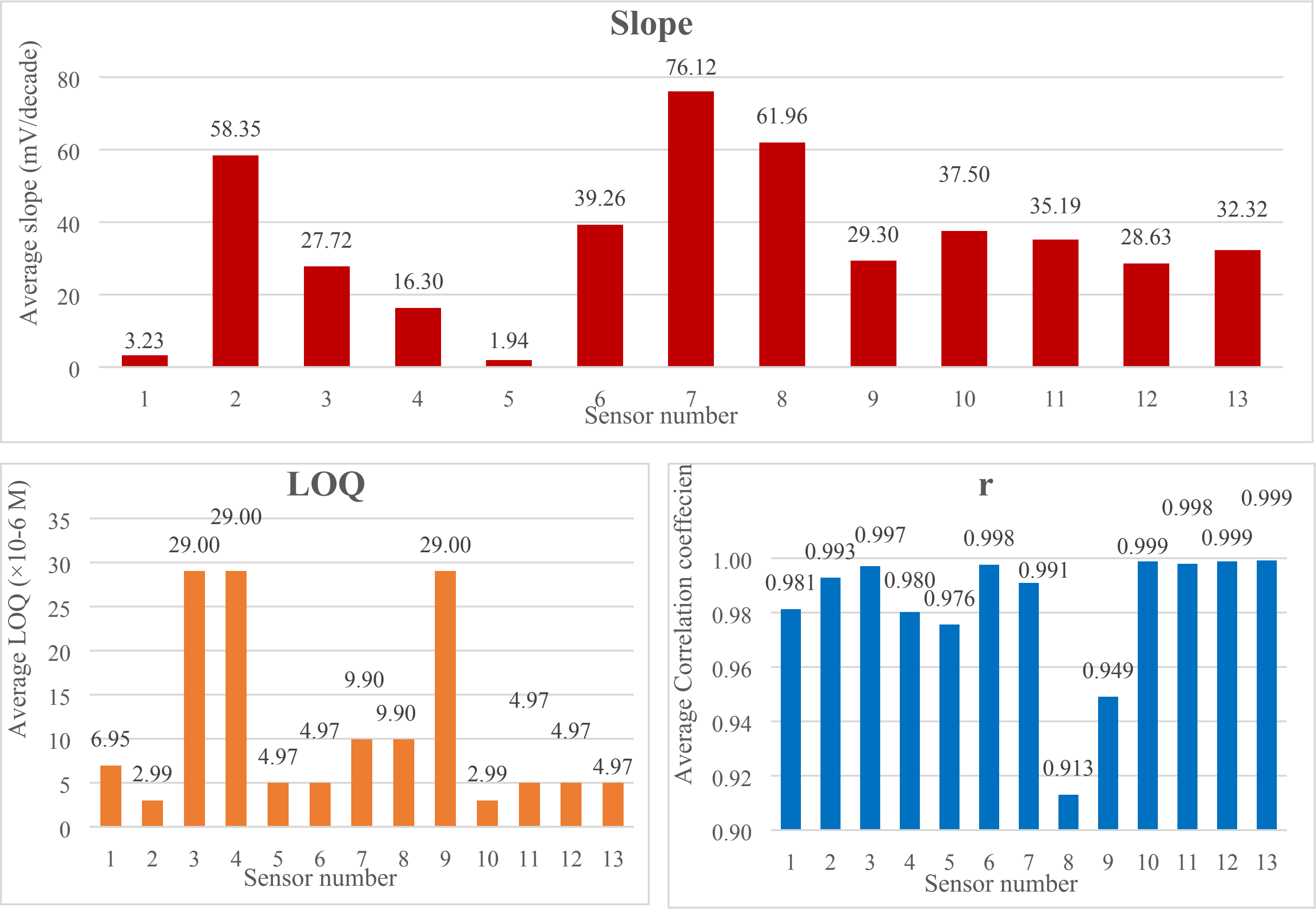

Supplementary Figure S2. The average response parameters—slope, LOQ, and correlation coefficient (R)—of the 13 studied sensors during the optimization study (n=3).

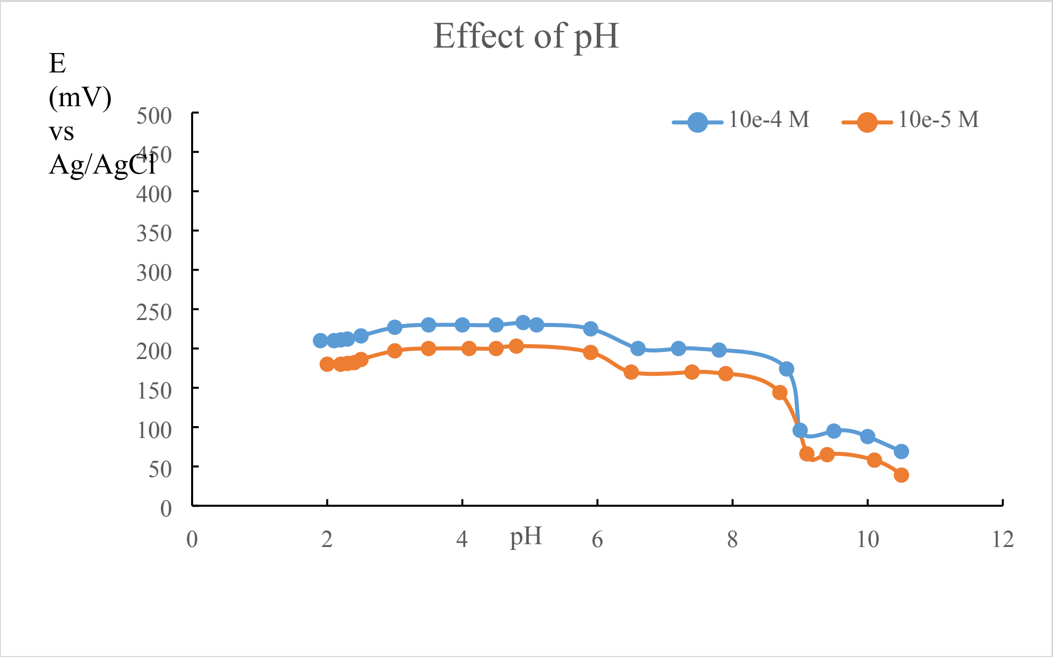

Supplementary Figure S3. Effect of pH on the potential response of the optimized sensor using $1{\times}10^{-4}$ and $1{\times}10^{-5}$ M vinblastine solutions.

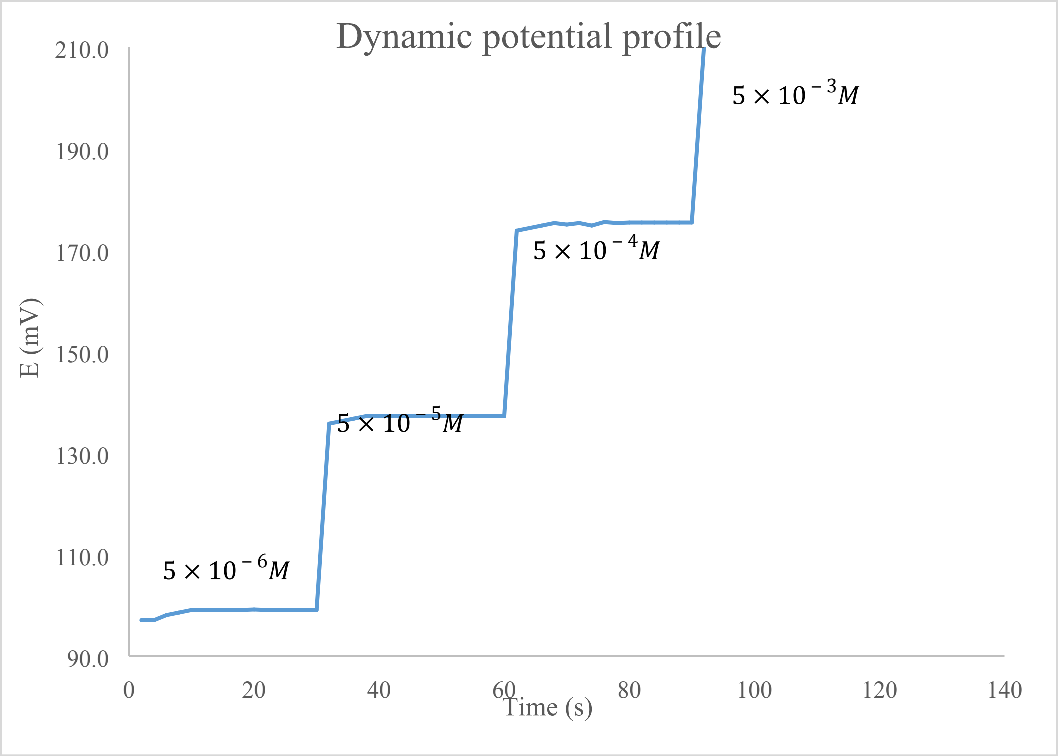

Supplementary Figure S4. Dynamic potential profile of the optimized sensor, the potential was recorded at two seconds sampling interval.

Supplementary Table S1. Composition of the studied sensors

Sensor no.	Sensor Composition (g% w/w)						
	PVC	Ion Exchanger		Plasticizer		Ionophore	
		Type	amount	Type	amount	Type	amount
Sensor 1	33.0 %	PM	1.0 %	NPOE	66.0 %	—	—
Sensor 2	33.0 %	PT	1.0 %	NPOE	66.0 %	—	—
Sensor 3	33.0 %	TPB	1.0 %	NPOE	66.0 %	—	—
Sensor 4	33.0 %	RK	1.0 %	NPOE	66.0 %	—	—
Sensor 5	33.0 %	TKS	1.0 %	NPOE	66.0 %	—	—
Sensor 6	33.0 %	TPB	1.0 %	DOP	66.0 %	—	—
Sensor 7	33.0 %	TPB	1.0 %	DBP	66.0 %	—	—
Sensor 8	33.0 %	TPB	1.0 %	DBS	66.0 %	—	—
Sensor 9	33.0 %	TPB	1.0 %	NPPE	66.0 %	—	—
**Sensor 10**	**32.3 %**	**TPB**	**1.0 %**	**DOP**	**64.7 %**	**HPBCD**	**2.0 %**
Sensor 11	32.3 %	TPB	1.0 %	DOP	64.7 %	BCD	2.0 %
Sensor 12	32.3 %	TPB	1.0 %	DOP	64.7 %	CMBCD	2.0 %
Sensor 13	32.3 %	TPB	1.0 %	DOP	64.7 %	CX8	2.0 %

Poly(vinyl)chloride (PVC), Phosphomolybdic acid (PM), phosphotungstic acid (PT), sodium tetraphenyl borate (TPB), ammonium reineckate (RK), potassium tetrakis (TKS), nitrophenyl octyl ether (NPOE), dioctyl phthalate (DOP), dibutyl phthalate (DBP), dibutyl sebacate (DBS), nitrophenyl phenyl ether (NPPE), hydroxypropyl-β-cyclodextrin (HPBCD), β-cyclodextrin (BCD), carboxymethyl-β-cyclodextrin (CMBCD), calix-[8]-arene (CX8).

Supplementary Table S2. The selectivity of the optimized sensor relative to cationic interfering species described as the potentiometric selectivity coefficient ($K_{VB,\,int}^{pot}$).

**Interfering Ion**	$K_{VB,\,int^{*}}^{pot}$
Vincristine	2.85×10^{-2}
Na^{+}	3.44×10^{-3}
K^{+}	3.23×10^{-3}
Zn^{2+}	3.23×10^{-3}
Mn^{2+}	2.87×10^{-3}
Pb^{2+}	3.65×10^{-3}
Co^{2+}	3.44×10^{-3}
Mg^{2+}	3.88×10^{-3}
Cu^{2+}	3.88×10^{-3}
Fe^{2+}	3.65×10^{-3}
Ca^{2+}	3.88×10^{-3}
Ni^{2+}	4.12×10^{-3}
NH_4^{+}	3.44×10^{-3}
Ba^{2+}	3.65×10^{-3}

* Determined by the separate solution method using 1×10^{-4} M solutions.

Supplementary Table S3. Application of the developed method for the determination of VB in Cytoblastin® vial and spiked plasma samples.

Application	Mean recovery ± %RSD*	Recovery of the standard added ± %RSD *
**Cytoblastin® Vial****	99.45 ± 1.101	101.50 ± 1.370
**Plasma**	100.03 ± 3.508	98.36 ± 3.780

* Average of five determinations

** Cytoblastin® aqueous injection for intravenous use only containing vinblastine sulfate 10mg/10mL, batch number 140573.
